# Urinalysis, Including Blanks for Recording the Analysis and Microscopic Examination of the Urine

**Published:** 1896-01

**Authors:** 


URINALYSIS, INCLUDING BLANKS FOR RECORDING THE ANALYSIS AND MICROSCOPIC EXAMINATION OF THE URINE.—For Medical Practitioners, Life Insurance Examiners and Specialists. Arranged by Joseph C. Guernsey, A. M., M. D. Philadelphia: J. B. Lippincott Company. 1895.

This is an octavo book of more than five hundred pages. The first part of the book contains a list of the necessary apparatus and chemical reagents for making urinary analyses, also directions for making these analyses, and a short article on diet suitable for persons suffering from diabetes and Bright's disease. The remainder of the book, some five hundred pages, is made up of blanks for the recording and preserving of the analyses. These pages of blanks are preceded by an index for the names of the patients.

This book will be a great convenience to the physician in the matter of keeping a record of the condition of the urine in each case, and these records are so arranged—being indexed—that they can easily be referred to at any time; thus the progress of the patient can be readily ascertained. The book is unique, eminently practical and useful, and so far as we know there is no other on the market like it. H.

